# Supplementary material for: The Effect of the ortho Nitro Group in the Solvolysis of Benzyl and Benzoyl Halides
Source: Int J Mol Sci. 2019 Aug 18;20(16):4026. doi: 10.3390/ijms20164026 (PMC6719289; doi:10.3390/ijms20164026)
Supplement: Supplementary file 1 [file ijms-20-04026-s001.pdf]

Supporting Materials

SM 1. Area percents (%) of *o*-nitrobenzoyl chloride and ethyl-*o*-nitrobenzoate (product) in 100% EtOH

Reat. time[a] (min.)	Area percent(%)	
	Ethyl-o-nitrobenzoate (5.3min)[b]	o-Nitrobenzoyl chloride (6.0min)[b]
1	42.6	57.4
11	83.9	16.1
21	97.4	2.6
31	100.0	-
41	100.0	-
150	100.0	-

[a]Reaction time. [b]Retention time

SM 2. Area percents (%) of o-nitrobenzoyl chloride, *o*-nitrobenzoic acid (product), and trifluoroethyl *o*-nitrobenzoate (product) in 70% TFE

Reat. time[a] (min.)	Area percent(%)		
	o-Nitrobenzoic acid (2.4min)	*o*-Nitrobenzoyl chloride (6.0min)	Trifluoroethyl *o*-nitrobenzoate (7.0min)
1	44.2	29.5	26.3

11	45.3	0.1	54.6
21	45.3	-	54.7
31	45.3	-	54.7

[a]Reaction time. [b]Retention time

SM 3. Analyzing conditions of HPLC for products by solvoyisis

Items	Analyzing conditions
System	Surveyor HPLC System (Thermo Finnigan Co. USA)
Detector	Surveyor PDA Detector
Pump	Finnigan Surveryor LC Pump
Column	150×4.6 ㎜ 5μ Hypersill® GOLD C18
Wavelength	325 ㎚
Solvent	ACN 50% : H_2O 50% Isocratic
Flow Rate	1.0 ㎖/min
Injection Volume	2 ㎕

SM 3. Analyzing conditions of GC/mass for identification of products by solvoylsis.

Items	Analyzing conditions

GC	System	Clarus 500 GC-Mass System (Perkin-Elmer, USA)
	column	Elite-5MS, (30mx0.32 mm IDx 0.25 ㎛ DF)
	carrier gas	He
	flow rate	1 ㎖/min
	Oven Temp	initial : 70℃, 2min, ramp : 10℃ min, 5min hold
	injection volume	1.0 ㎕
	split ratio	50 : 1
Mass	Ion mode	EI
	mass range	30 amu ~ 500 amu
	solvent D. time	2 min

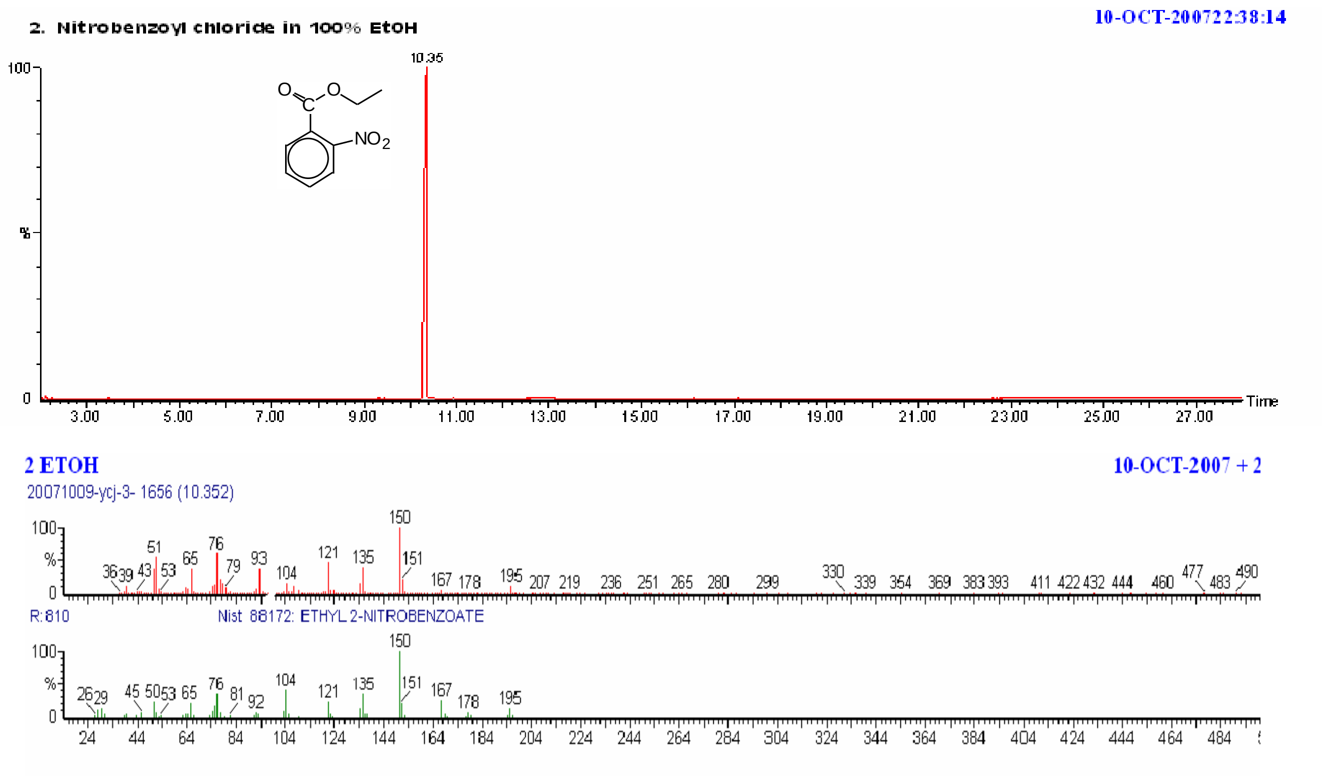

SM 4. GC/mass total ion chromatogram and mass spectrum of product of *o*-nitrobenzoyl chloride in 100% ethanol.

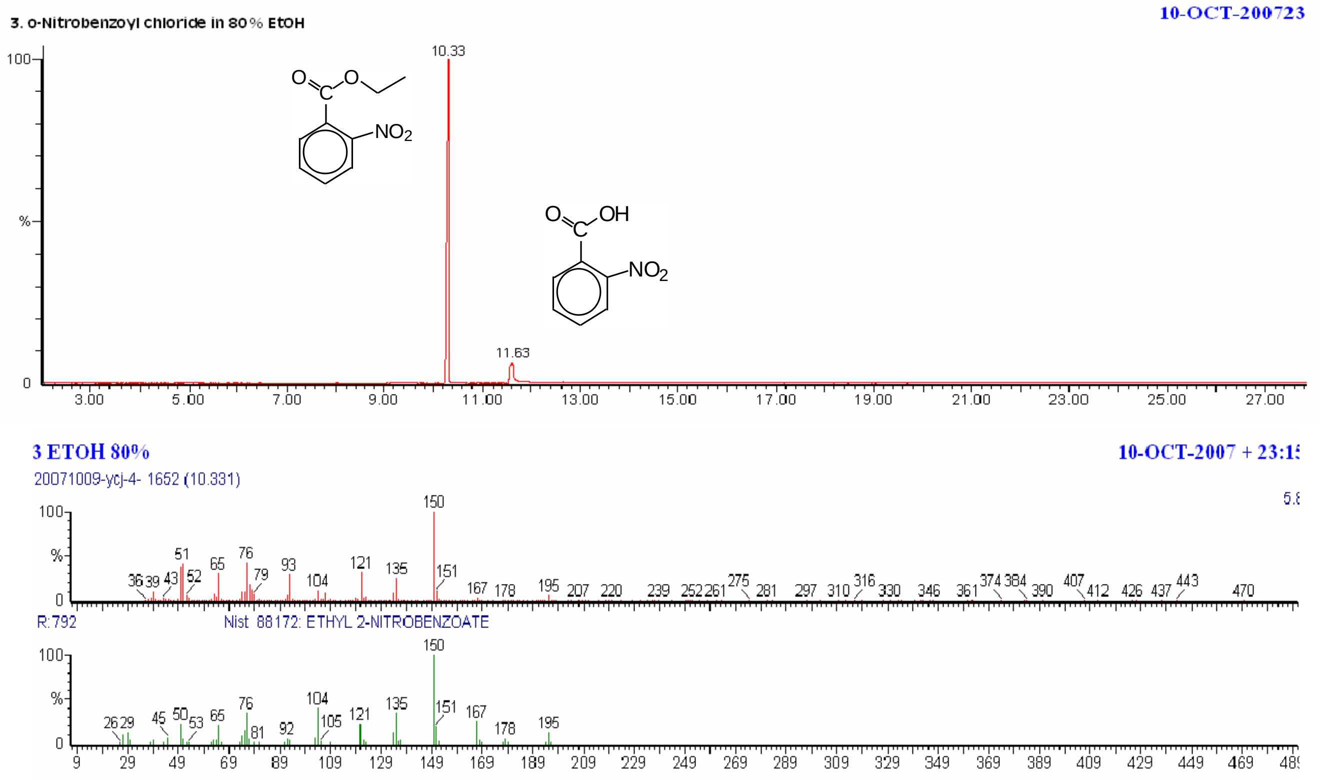

SM 5. GC/mass total ion chromatogram and mass spectrum of product of *o*-nitrobenzoyl chloride in 80% aqueous ethanol at 10.33 min.

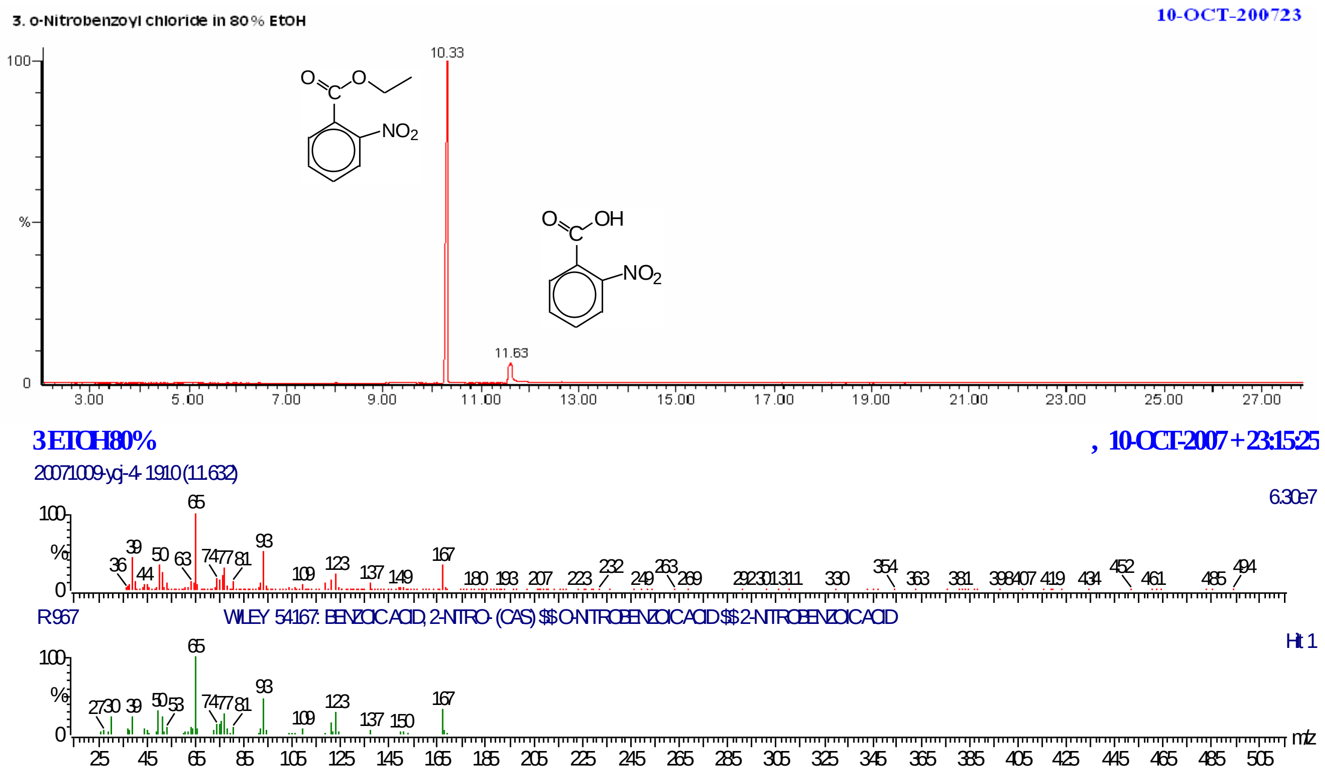

SM 6. GC/mass total ion chromatogram and mass spectrum of product of *o*-nitrobenzoyl chloride in 80% aqueous EtOH at 11.63 min.

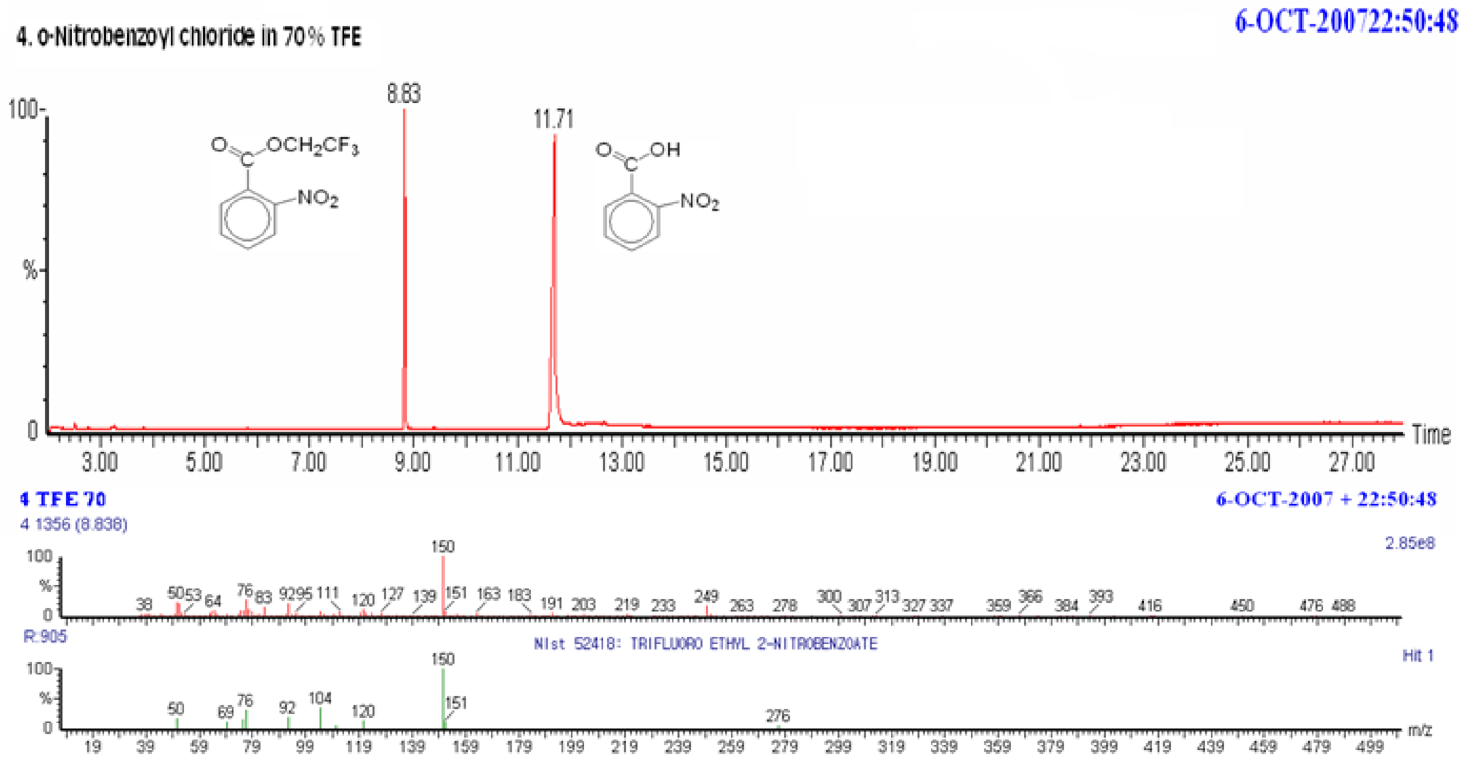

SM 7. GC/mass total ion chromatogram and mass spectrum of product of *o*-nitrobenzoyl chloride

in 60% aqueous TFE.

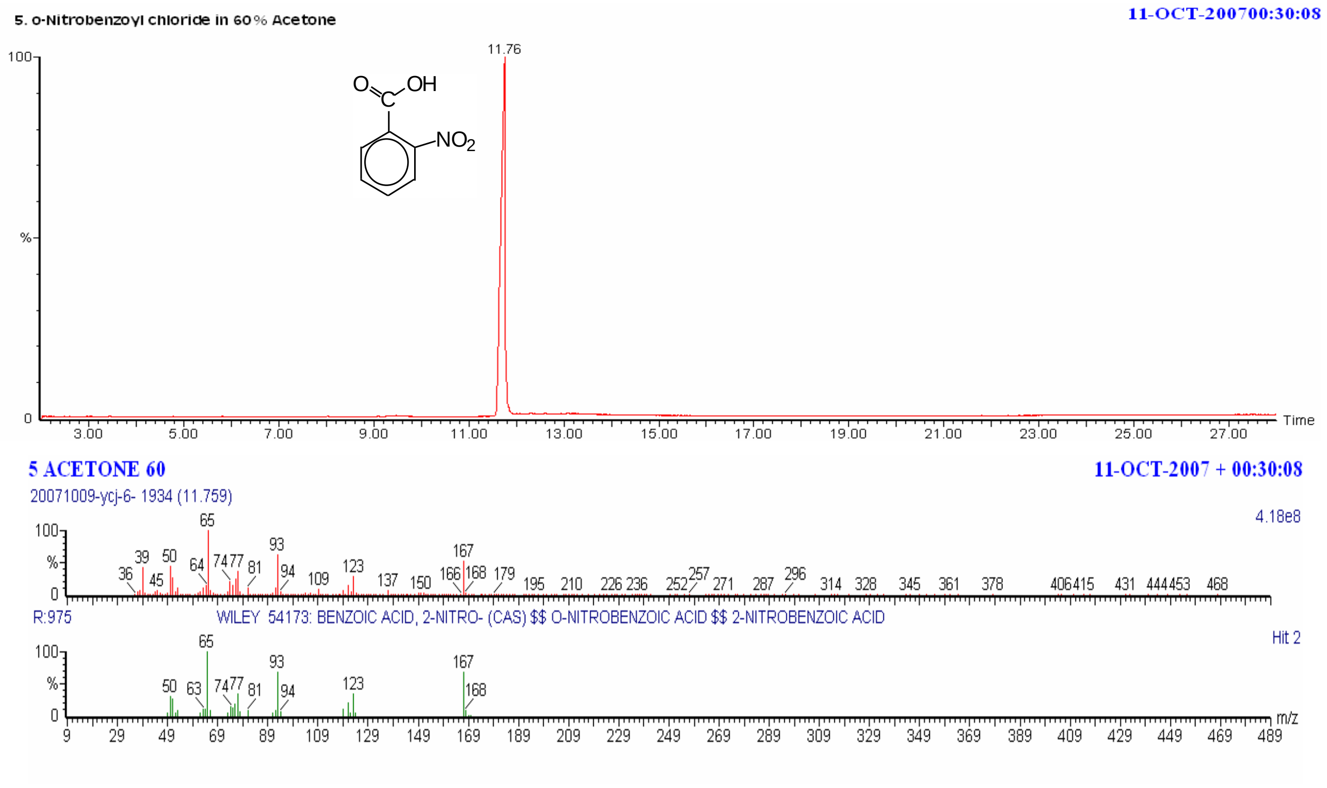

SM 8. GC/mass total ion chromatogram and mass spectrum of product of *o*-nitrobenzoyl chloride in 60% aqueous acetone.

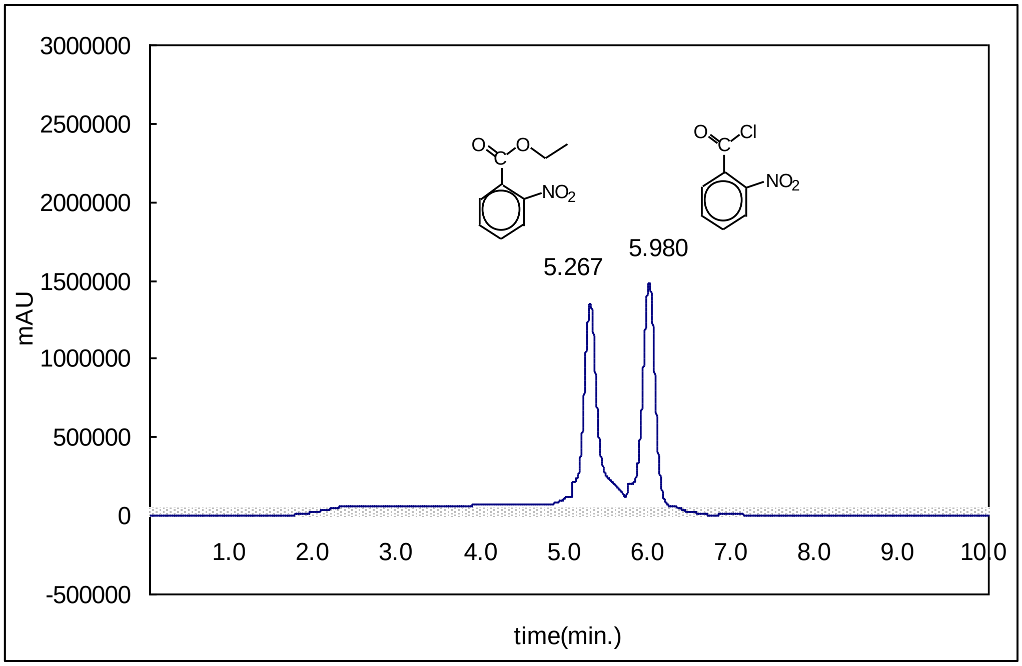

SM 9. HPLC chromatogram of *o*-nitrobenzoyl chloride in 100%EtOH at 1 minute.

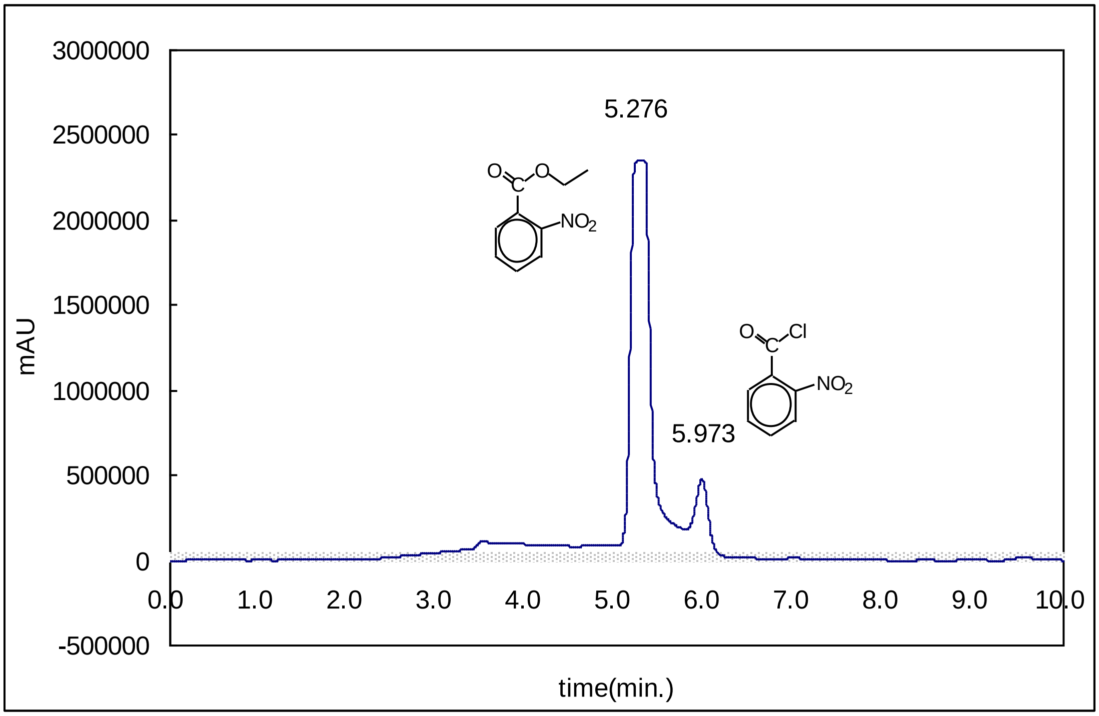

SM 10. HPLC chromatogram of *o*-nitrobenzoyl chloride in 100%EtOH at 11 minute.

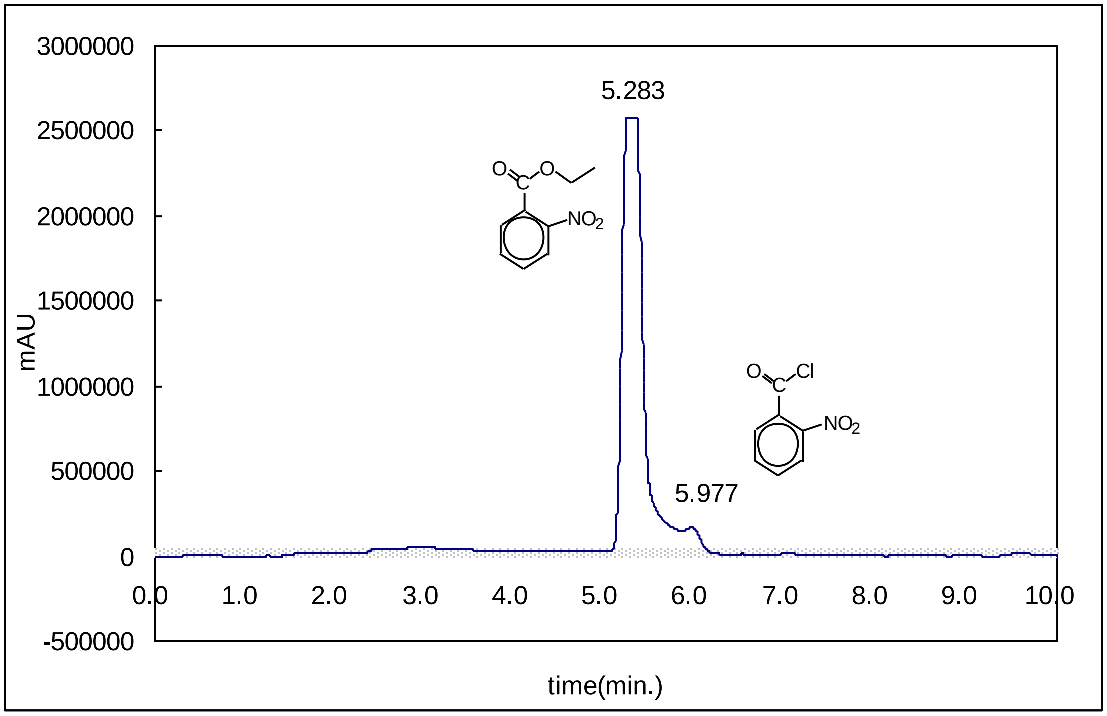

SM 11. HPLC chromatogram of *o*-nitrobenzoyl chloride in 100%EtOH at 21 minute.

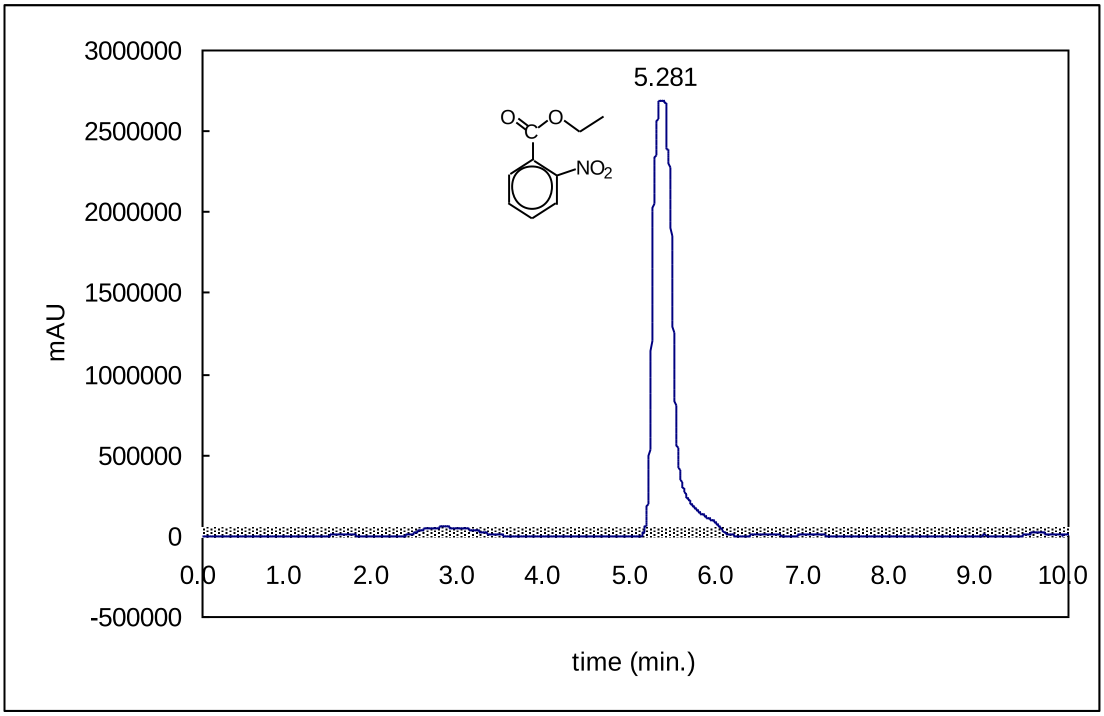

SM 12. HPLC chromatogram of *o*-nitrobenzoyl chloride in 100%EtOH at 150 minute.

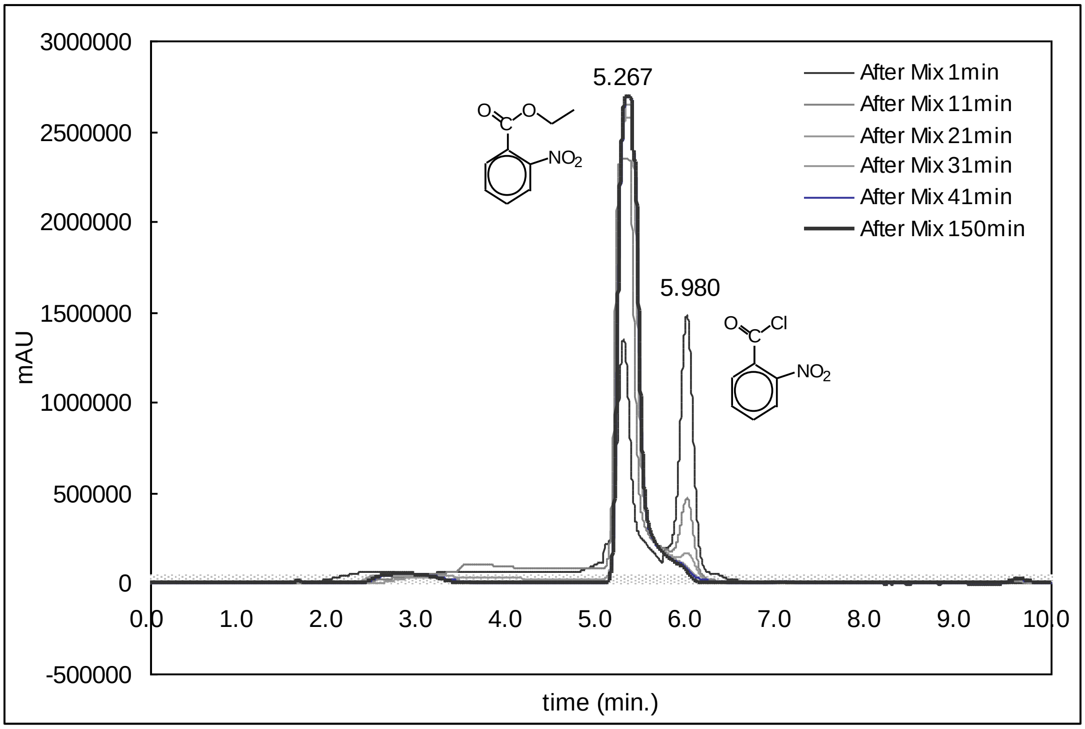

SM 13. HPLC chromatogram of *o*-nitrobenzoyl chloride in 100%EtOH at various times.

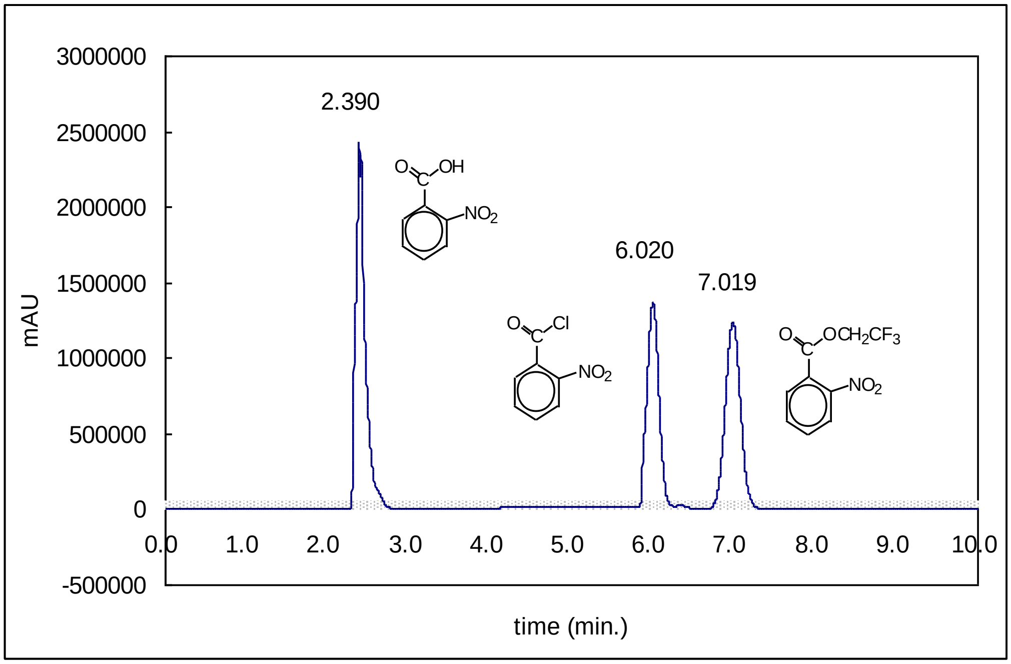

SM 14. HPLC chromatogram of *o*-nitrobenzoyl chloride in 70%TFE at 1 minute.

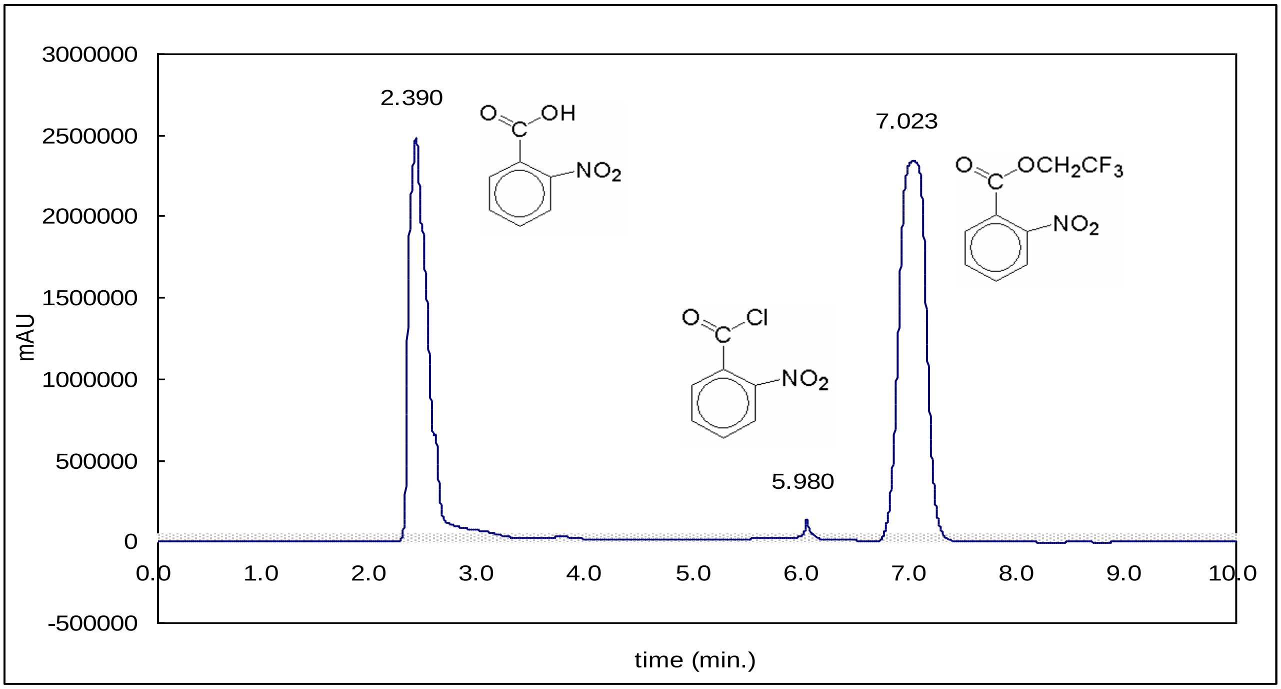

SM 15. HPLC chromatogram of o-nitrobenzoyl chloride in 70%TFE at 11 mnute.

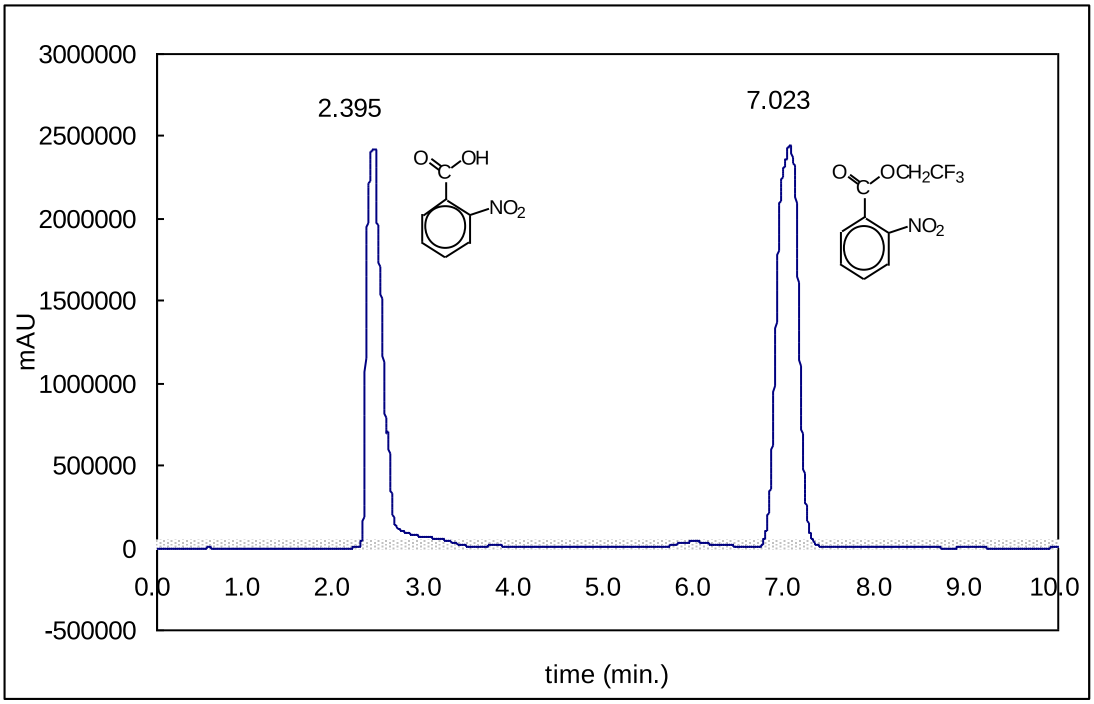

SM 16. HPLC chromatogram of *o*-nitrobenzoyl chloride in 70%TFE at 31 minute.

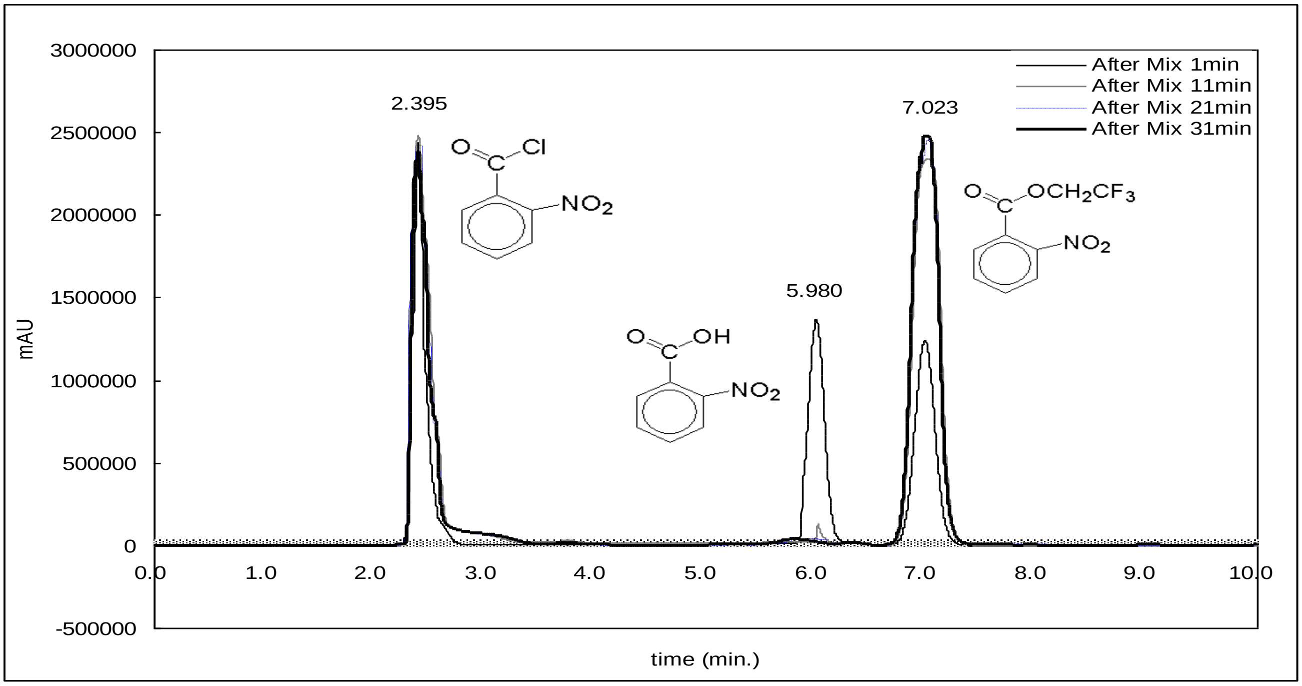

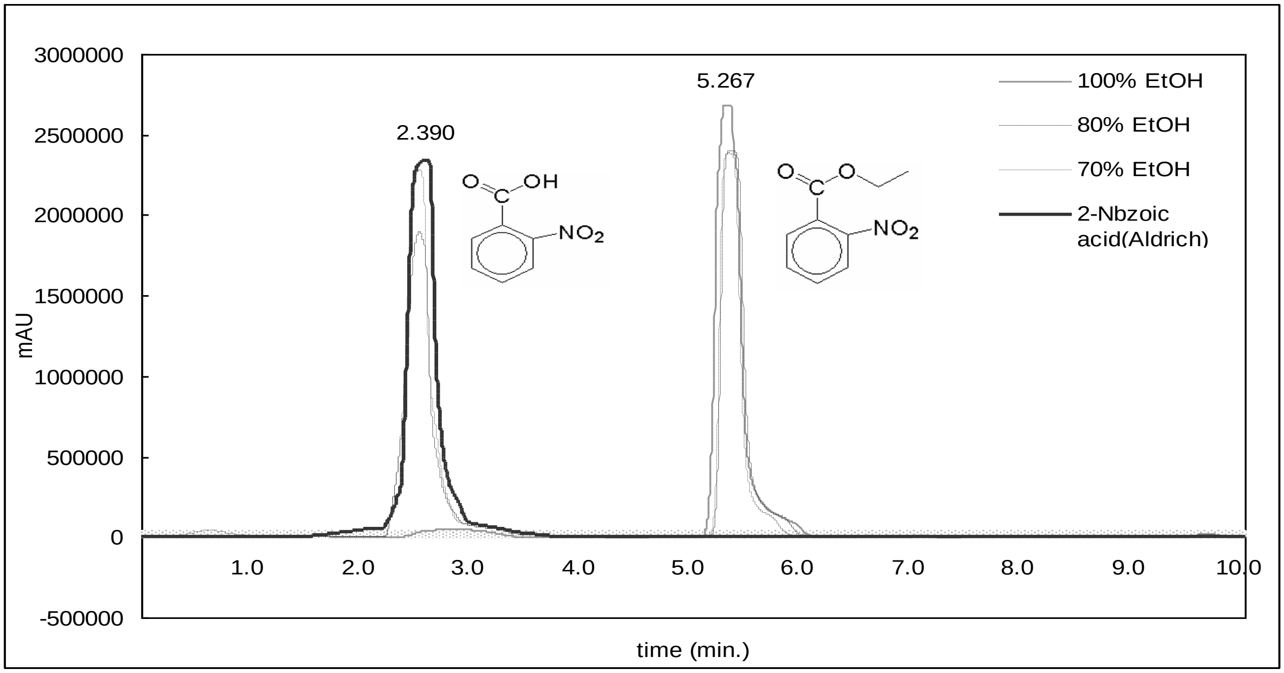

SM 17. HPLC chromatogram of *o*-nitrobenzoyl chloride in various concentrations of pure and aqueous ethanol

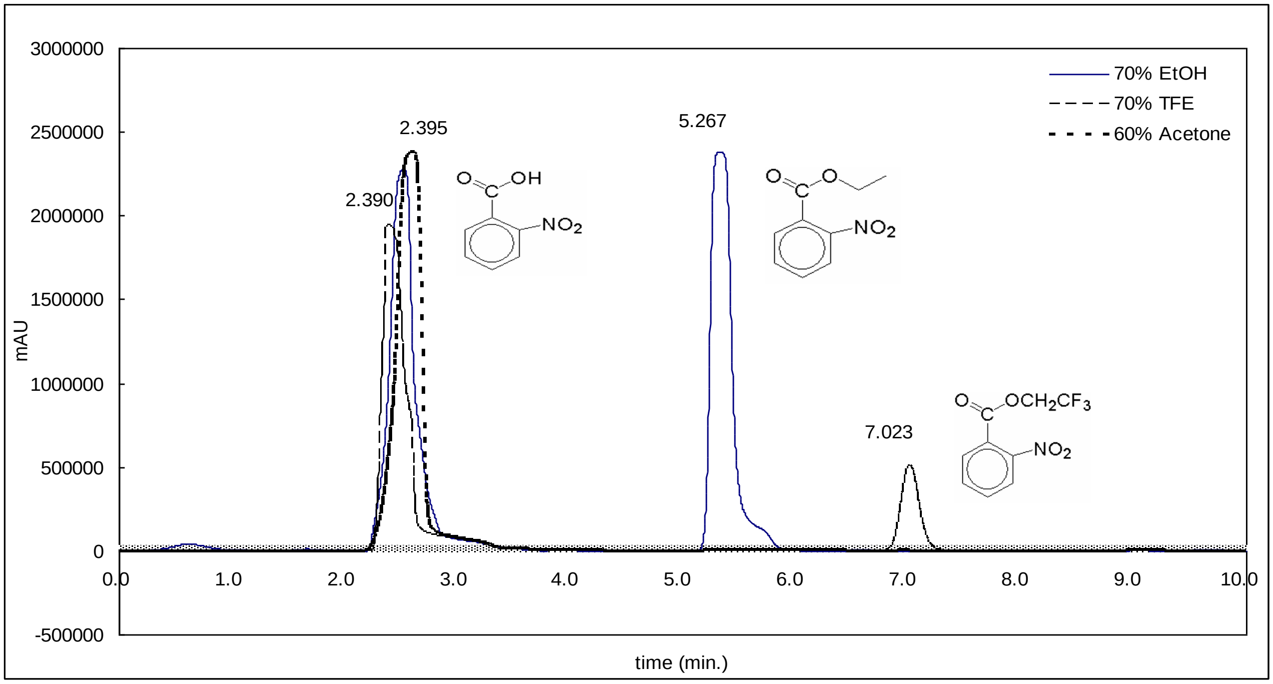

SM 18. HPLC chromatogram of *o*-nitrobenzoyl chloride in various aqueous solvents.
